# ZmDLR9 is required for lateral root development via pre‐rRNA processing in maize

**DOI:** 10.1111/tpj.71156

**Published:** 2026-10-08

**Authors:** Daojun Li, Yang Li, Aiming Xing, Shixian Zhang, Yanbin Zhu, Feng Wei, Haitao Zeng, Tao Zheng, Zhubing Hu, Chunfei Wang

**Affiliations:** ^1^ State Key Laboratory of Crop Stress Adaptation and Improvement, School of Life Sciences Henan University Kaifeng 475004 China; ^2^ School of Biological Science and Engineering Shaanxi University of Technology Hanzhong Shaanxi 723001 China; ^3^ Sanya Institute of Henan University Sanya 572025 China; ^4^ Hainan Agricultural Reclamation Academy of Sciences Group Co., Ltd. Sanya 570105 Hainan China; ^5^ Xinxiang Academy of Agricultural Sciences Xinxiang 453000 China

**Keywords:** lateral root, Pumilio, ZmDLR9, pre‐rRNA processing, cell proliferation

## Abstract

Pumilio (PUM) proteins comprise an evolutionarily conserved family of sequence‐specific RNA‐binding proteins that act as key post‐transcriptional regulators in eukaryotes. In this study, we identified a maize PUM protein, named ZmDLR9 (Defective in Lateral Root 9), as a positive factor involved in lateral root development. Loss‐of‐function mutation in the *ZmDLR9* (*dlr9‐1*) severely impaired lateral root formation. Functional analysis revealed that ZmDLR9 localizes in the nucleolus and interacts with pre‐rRNA processing‐associated proteins, including BRX1‐1 (BIOGENESIS OF RIBOSOMES IN XENOPUS 1–1), BRX1‐2, and GRC3 (a subunit of the ITS2 processing complex). The *dlr9‐1* mutation resulted in aberrant accumulation of pre‐rRNA processing intermediates, as well as defects in ribosome assembly and translation. Heterologous expression of *ZmDLR9* partially rescued the growth defects of an Arabidopsis PUM loss‐of‐function mutant (*apum24‐2*), demonstrating functional conservation of the PUM protein family across the plant kingdom. Furthermore, the *dlr9‐1* mutation disrupted nucleolar morphology, thereby inhibiting cell proliferation and delaying lateral root primordia emergence. Together, our results demonstrate that ZmDLR9, a Pumilio protein, is required for lateral root development by mediating pre‐rRNA processing‐dependent cell proliferation.

## Significance Statement

Lateral root development is critical for nutrient uptake in cereal crops, yet its post‐transcriptional regulation remains poorly understood. We show that the maize PUM protein ZmDLR9 is involved in cell proliferation by mediating pre‐rRNA processing and nucleolar integrity, thereby directing lateral root formation, a distinct mechanism that provides a potential target for improving root architecture.

## INTRODUCTION

The Pumilio (PUM) protein contains a conserved RNA‐binding domain known as Pumilio homology domain, which is evolutionarily conserved across eukaryotes. In mammals, PUM proteins bind a wide range of RNAs and repress gene expression by inhibiting translation and promoting mRNA decay, playing essential roles in stem cell fate, development, and neurological functions (Goldstrohm et al., 2018). The expression and function of PUM proteins are finely regulated by regulatory factors such as miRNAs, lncRNAs, and circRNAs, and they play important roles in tumorigenesis through various regulatory axes (Silva et al., 2022). Notably, beyond upstream regulation, excessive PUM proteins suppress the stability and translation of their target mRNAs, thereby inhibiting cellular mitosis (Lee et al., 2016). For example, in mice, Pum1 and Pum2 form a negative feedback loop that regulates over 1400 mRNAs via translational repression/activation and mRNA stability control, exerting essential and complementary functions in embryonic stem cell (ESC) pluripotency and embryogenesis, with Pum1 promoting differentiation and Pum2 promoting self‐renewal (Uyhazi et al., 2020). In human cells, PUM proteins bind 3′UTRs to promote mRNA decay (Farberov & Ulitsky, 2024).

Compared with the well‐characterized functions of PUM proteins in animals, the roles of their plant counterparts remain relatively underexplored. In Arabidopsis, 26 PUM genes have been identified, among which a few have been functionally characterized (Feng et al., 2023). These studies reveal that plant PUM proteins are involved in diverse biological processes, including viral defense, ribosome biogenesis, and embryonic development (Abbasi et al., 2010; Huh et al., 2013; Huh & Paek, 2014; Maekawa et al., 2018; Shanmugam et al., 2017). In viral defense, Arabidopsis Pumilio protein 5 (APUM5) suppresses Cucumber mosaic virus (CMV) infection by directly binding to specific motifs in the 3′UTR and internal regions of CMV RNAs, thereby acting as a translational repressor to inhibit viral replication (Huh et al., 2013; Huh & Paek, 2014). In ribosome biogenesis, two PUF family proteins, APUM23 and APUM24, play distinct yet complementary roles (Maekawa et al., 2018; Zhang & Muench, 2015). APUM23 facilitates the early cleavage of 35S pre‐rRNA and promotes the poly(A)‐dependent degradation of unprocessed rRNA precursors (Abbasi et al., 2010). It recognizes a specific 10‐nucleotide motif (GAAUUGACGG) within 18S rRNA to ensure accurate 18S rRNA maturation, a process essential for leaf development and organ polarity (Zhang & Muench, 2015). In contrast, APUM24 participates in later steps of rRNA processing, particularly in the removal of ITS2 from 5.8S rRNA, and interacts with pre‐rRNA processing factors such as BRX1‐1, BRX1‐2, AtLAS1, and AtGRC3 (Maekawa et al., 2018; Shanmugam et al., 2017). Loss of APUM24 disrupts ribosome assembly, induces nucleolar stress, and impairs embryonic patterning by mislocalizing PIN1 and PIN7 auxin transporters (Maekawa et al., 2018; Shanmugam et al., 2017). Beyond rRNA processing, APUM24 also post‐transcriptionally regulates BPM family mRNA stability, thereby stabilizing WRINKLED1 and promoting seed oil accumulation (Huang et al., 2021). Despite these advances in Arabidopsis, the functional characterization of maize PUM homologs remains limited.

In this study, we identified a maize homolog of APUM24, named ZmDLR9 (Defective in Lateral Root 9). ZmDLR9 interacts with proteins involved in pre‐rRNA processing. Loss‐of‐function mutation of ZmDLR9 (i.e., the *dlr9‐1* allele) disrupts pre‐rRNA processing, causes nucleolar abnormalities, and activates a nucleolar stress response that suppresses cell proliferation, ultimately impairing lateral root development. These findings establish a novel link between PUM‐mediated rRNA processing and lateral root development in maize, providing insights into the molecular mechanisms that underlie root architecture formation in cereal crops.

## RESULTS

### ZmDLR9 regulates lateral root development in maize

A lateral root‐defective mutant, designated *dlr9‐1*, was isolated from an ethyl methanesulfonate (EMS)‐mutagenized population of the maize inbred line B73. Compared with wild‐type B73, *dlr9‐1* showed a significant reduction in lateral root (LR) number, whereas primary root (PR) length remained unchanged (Figure 1a; Figure S1A–D). After 7 days of hydroponic cultivation, LRs were rarely observed in *dlr9‐1* seedlings (Figure S1A,B). Because LR emergence depends on the penetration of LR primordia (LRP) through the epidermis, we further quantified LRP numbers in B73 and *dlr9‐1* seedlings using Feulgen staining. Notably, no significant difference in LRP number was found between B73 and *dlr9‐1* (Figure S1E,F). Furthermore, we statistically compared the size of LR primordia between B73 and *dlr9‐1* using paraffin sections. This analysis revealed that *dlr9‐1* primordia were significantly smaller and predominantly arrested at the S1–S3 stages, whereas B73 primordia had already emerged through the epidermis (Figure S2). This suggests that impaired LR development in *dlr9‐1* does not occur at the early initiation stage of LRP, but rather stems from delayed LRP development at later stages.

**Figure 1:**
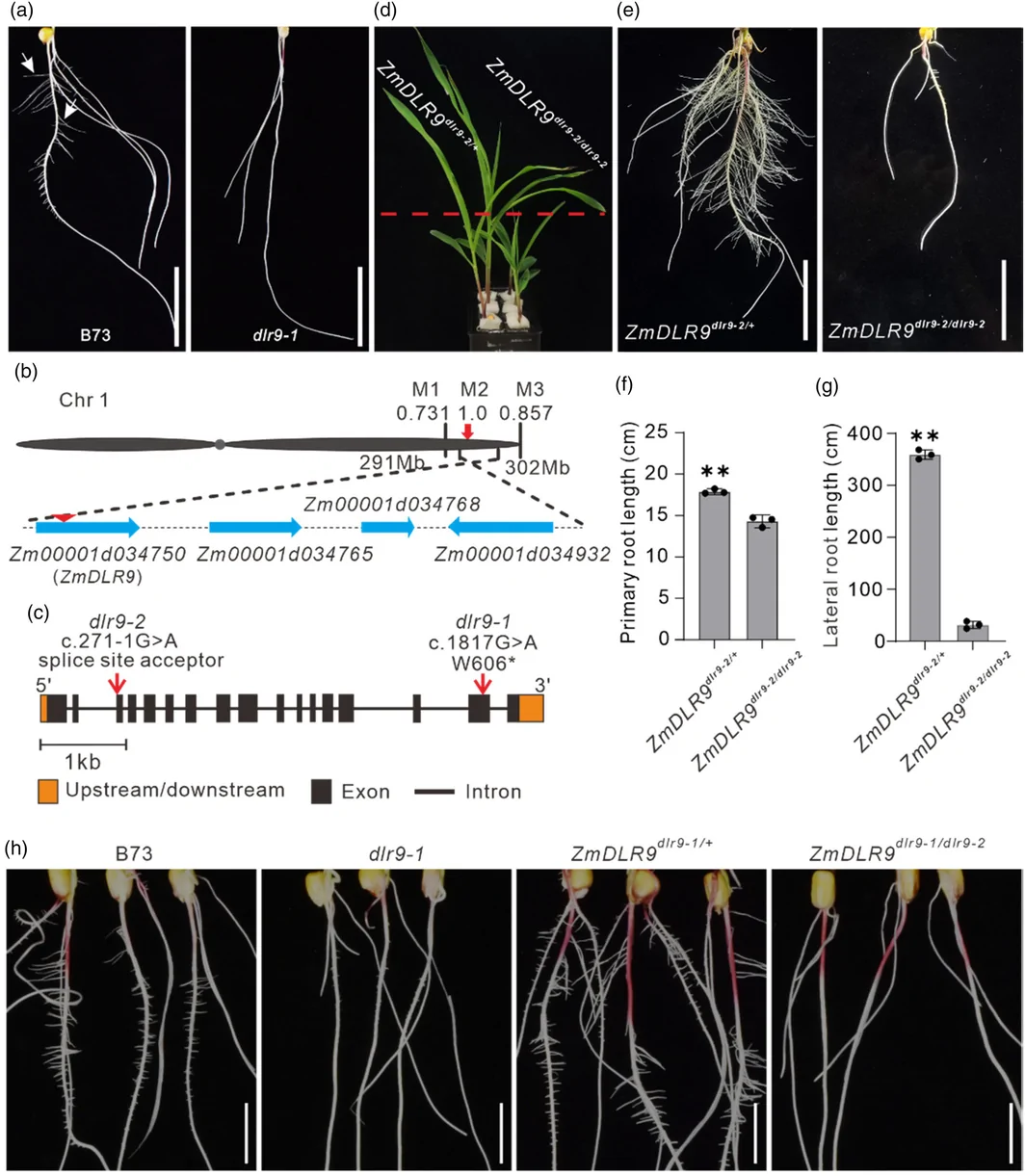
ZmDLR9 regulated lateral root development. (a) Root morphology of B73 and *dlr9‐1* seedlings at 6 days after germination. The white arrow indicates lateral root. Scale bar = 5 cm. (b) Map‐based cloning of *ZmDLR9*. *ZmDLR9* was mapped between markers M1 and M3 on chromosome 1. Numbers below the molecular marker indicate recombinant rates in *dlr9‐1*/B73 F_2_ population. (c) Gene model of *ZmDLR9*. Black boxes indicate exons, black lines indicate introns, and yellow boxes indicate the 5′ or 3′ untranslated regions. The red arrows indicate mutation sites of *dlr9‐1* and *dlr9‐2* in *ZmDLR9* gene, respectively. (d) Seedlings of heterozygous (*ZmDLR9* ^*dlr9‐2/+*^, left) and homozygous (*ZmDLR9* ^*dlr9‐2/dlr9‐2*^, right) plants grown under hydroponic conditions. Scale bar = 5 cm. (e) Root architecture of *ZmDLR9* ^*dlr9‐2/+*^ and *ZmDLR9* ^*dlr9‐2/dlr9‐2*^. Scale bar = 5 cm. (f, g) Quantitative analysis of primary root length (F) and lateral root length (G) of *ZmDLR9* ^*dlr9‐2/+*^ and *ZmDLR9* ^*dlr9‐2/dlr9‐2*^. Data are presented as mean ± SD (*n* = 5). Asterisks indicate significant differences (*P* < 0.01, Student's *t*‐test). (h) Root phenotypes of F_1_ progeny and parental seedlings. Scale bar = 2 cm.

To identify the causal gene responsible for the LR developmental defect, we backcrossed *dlr9‐1* with B73 to generate F_1_ progeny. All F_1_ individuals exhibited normal primary and LR phenotypes, indicating that the mutation is inherited recessively (Figure S3A). Self‐pollination of F_1_ plants produced an F_2_ population that segregated into two phenotypic classes: seedlings with B73‐like roots and those with *dlr9‐1*‐like roots, which phenocopied the parental *dlr9‐1* mutant (Figure S3A). Both *dlr9‐1*‐like F_2_ seedlings and the parental *dlr9‐1* showed significantly reduced LR numbers compared with B73 (Figure S3B), whereas no statistically significant difference was observed in their PR length (Figure S3C). The F_2_ progeny exhibited an approximate 3:1 segregation ratio (B73‐like: *dlr9‐1*‐like) (Figure S3A; Table S1), confirming that *dlr9‐1* is a recessive nuclear monogenic mutation.

To rapidly identify the candidate gene (designated *ZmDLR9*), we performed bulk segregant analysis sequencing (BSA‐seq) using leaf tissues pooled from more than 30 *dlr9‐1‐like* F_2_ seedlings. Equal amounts of leaf tissue were combined for DNA extraction, followed by whole‐genome resequencing. Comparative analysis against the B73 V4 reference genome identified single‐nucleotide polymorphism (SNP) variants. Subsequent MutMap analysis revealed a prominent SNP index peak at the terminal region of chromosome 1 (Figure S4A,B), thereby localizing the genetic locus responsible for *dlr9‐1* phenotype to this interval. Sliding window analysis narrowed the candidate interval to 6.09 Mb segment (positions 300 944 916–307 039 376 bp) near the chromosome terminus, where SNPs displayed SNP index values greater than 0.8 (Table S2). We then comprehensively screened all mutation sites within this interval (Table S3) and identified 28 SNPs with a frequency exceeding 0.8 (Table S4). All of these were G‐to‐A or C‐to‐T transitions, consistent with EMS‐induced mutagenesis (Table S4). Functional annotation categorized these variants into distinct genomic regions: one exonic variant, one in the 5′ untranslated region (UTR), one in a downstream gene element, two intronic variants, and the majority in intergenic regions (Table S4). Notably, the exonic variant within *Zm00001d034750* introduces a premature termination codon (Figure 1b,c; Table S4), which is predicted to result in a truncated protein product. As the other variants lacked clear strong functional impacts, we prioritized *Zm00001d034750* as the most promising candidate gene for *ZmDLR9*.

To validate *ZmDLR9* as the causal gene, we obtained an independent EMS‐induced allele (EMS4‐0 dc597) from a B73 EMS‐mutagenized library (Lu et al., 2018) and designated it *dlr9‐2* (Figure S5). The *dlr9‐2* allele carries a G‐to‐A mutation at the 3′ end of the second intron of *Zm00001d034750*, which disrupts the intron acceptor splice site (Figure 1c; Figures S5 and S6A). Phenotypic analysis showed that homozygous *dlr9‐2* seedlings exhibited severe developmental defects in both primary and LRs (Figure 1d,e). To assess whether this splice‐site mutation impairs splicing of the second intron, we extracted total RNA from B73 and *dlr9‐2* seedlings, reverse‐transcribed it into cDNA, and amplified the region flanking the second intron using specific primers (Figure S6A). Amplification products from *dlr9‐2* were larger than those from B73 (Figure S6B,C), suggesting possible intron retention due to aberrant splicing. Sanger sequencing confirmed that the second intron was correctly spliced in B73, whereas it was retained in *dlr9‐2* (Figure S6D,E), a defect predicted to cause premature translational termination.

To confirm that *dlr9‐1* and *dlr9‐2* are allelic, we performed a complementation test by crossing homozygous *dlr9‐1* (*ZmDLR9ᵈˡʳ* ^*9‐1*^ */ᵈˡʳ* ^*9‐1*^) with heterozygous *dlr9‐2* (*ZmDLR9ᵈˡʳ* ^*9‐2*^ */* ^*+*^) (Figure S7A). The F_1_ progeny segregated into two phenotypic classes: B73‐like seedling with normal LR development and *dlr9‐1‐*like seedlings with LR defects that phenocopied the parental *dlr9‐1* mutant, at a ratio of 56:46 (Figure 1h; Figure S7A–D), which approximates a 1:1 segregation pattern (Figure S7A). To establish genotype–phenotype co‐segregation, we genotyped these F_1_ seedlings. Sequencing results showed that all F_1_ individuals with B73‐like phenotypes were heterozygous (*ZmDLR9ᵈˡʳ* ^*9‐1*^ */* ^*+*^), whereas those with LR defects were trans‐heterozygous for the mutant alleles (*ZmDLR9ᵈˡʳ* ^*9‐1*^ */ᵈˡʳ* ^*9‐2*^) (Figure S7E). This complete linkage between genotype and phenotype confirmed the genetic basis of trait segregation. These results demonstrate that *dlr9‐2* heterozygosity fails to rescue the LR defect of *dlr9‐1* mutants (Figure 1h). Moreover, the co‐segregation of mutation sites with phenotype at an approximately 1:1 ratio supports a single‐gene inheritance pattern (Figure S7A). Collectively, this genetic evidence confirms that *ZmDLR9* is the causal gene regulating LR development in maize.

### ZmDLR9 is required for efficient pre‐rRNA processing

To predict the functional characteristics of ZmDLR9, we performed motif analysis using the SMART platform (https://smart.embl.de/) (Letunic et al., 2021). The results showed that ZmDLR9 belongs to the Pumilio protein family and contains four Pumilio motifs (Figure S9B). Given that *Arabidopsis thaliana* is the most well‐characterized species in plant functional genomics, we conducted genomic surveys in maize and Arabidopsis, identifying 19 and 25 PUM genes, respectively. A phylogenetic tree constructed with maize and Arabidopsis PUM proteins resolved into four distinct clades, with ZmDLR9 and APUM24 clustering together in Clade II (Figure S8), indicating that ZmDLR9 is the maize homolog of APUM24. We then obtained APUM24 homologs from *Zea mays*, *Sorghum bicolor*, *Setaria italica*, *Nicotiana tabacum*, *Populus euphratica*, *Oryza sativa*, and *Arabidopsis thaliana*, and performed sequence alignment and motif analysis. All selected homologs exhibited high conservation, with over 47% identity to ZmDLR9, and contained 4–5 conserved Pumilio (PUM) motifs (Figure S9A,B).

To dissect how *ZmDLR9* regulates LR development, we analyzed its tissue‐specific expression at the transcript level and its subcellular localization at the protein level. *ZmDLR9* was highly expressed in young roots, young leaves, and silks, while its expression levels were relatively low in ears and tassels (Figure 2a). To determine the subcellular localization of ZmDLR9, we first analyzed the major transcripts in B73 seedling roots and identified *ZmDLR9_T003* as the predominant transcript (Figure S10). Subsequently, we generated a construct fusing ZmDLR9 to red fluorescent protein (RFP) at its C‐terminus and performed co‐localization assays in *Nicotiana benthamiana* leaves using the Arabidopsis nucleolar marker AtFIB1‐GFP. Confocal microscopy showed that ZmDLR9‐RFP signals overlapped completely with AtFIB1‐GFP in the nucleolus (Figure 2b). To validate this localization in maize, we repeated the co‐localization assay in maize leaf protoplasts: ZmDLR9‐RFP again co‐localized with AtFIB1‐GFP (Figure 2c), confirming that ZmDLR9 is a nucleolar protein.

**Figure 2:**
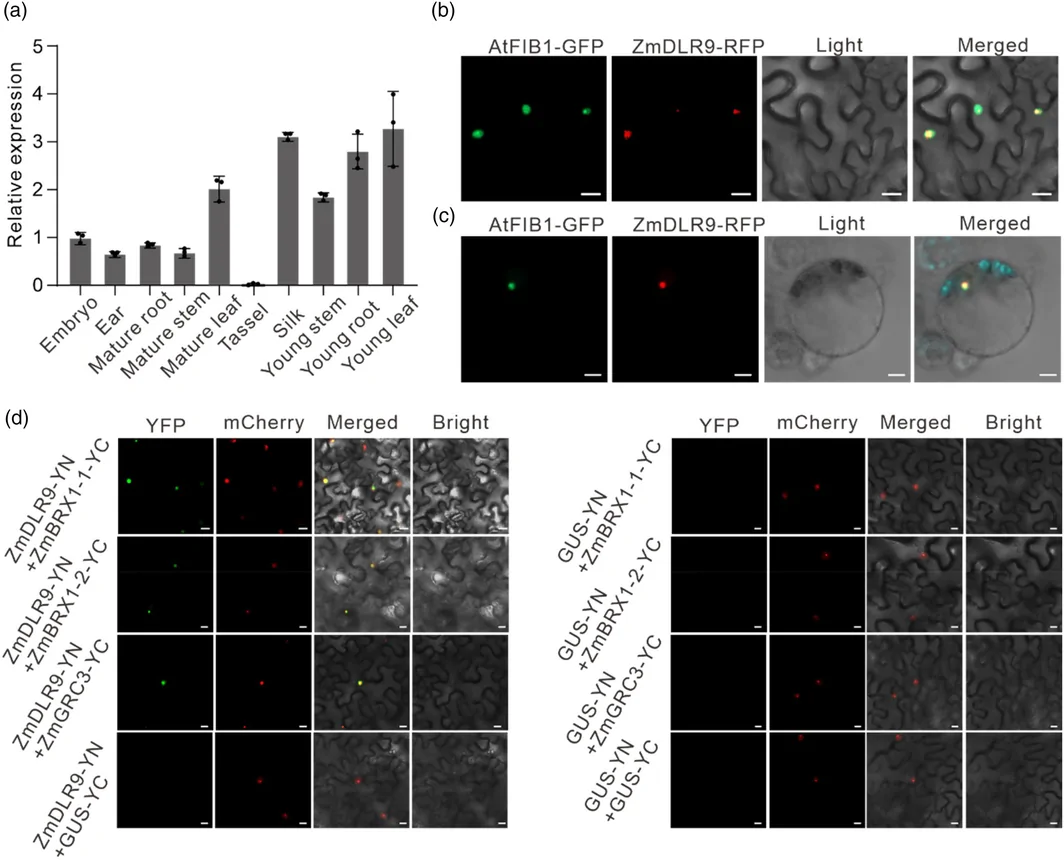
Expression pattern, subcellular localization, and interaction with pre‐rRNA processing‐related proteins of ZmDLR9. (a) Quantitative RT‐PCR of *ZmDLR9* expression from different tissues of B73. Datas are presented as mean ± SD (*n* = 3). (b) Subcellular localization of ZmDLR9 protein in *N. benthamiana*. AtFIB1‐GFP signal was used as a nuclear reporter. The merged image demonstrates the nuclear localization of ZmDLR9‐RFP Scale bar = 20 μm. (c) Subcellular localization of ZmDLR9 protein in maize protoplasts. Scale bar = 5 μm. (d) Interaction between ZmBRX1‐1, ZmBRX1‐2 or ZmGRC3 with ZmDLR9 via bimolecular fluorescence complementation (BiFC) assays in *N. benthamiana*. YC, C‐terminus of YFP; YN, N terminus of YFP. Scale bar = 10 μm.

In Arabidopsis, APUM24 is likely an ITS2 removal‐associated factor that interacts with pre‐rRNA processing‐associated proteins in the nucleolus, including BIOGENESIS OF RIBOSOMES IN XENOPUS 1–1 (BRX1‐1), its paralog BRX1‐2, and GRC3, which are subunits of the ITS2 processing complex (Maekawa et al., 2018). To investigate whether ZmDLR9, the maize ortholog of APUM24, functions in pre‐rRNA processing, we first performed BiFC assays in *Nicotiana benthamiana* leaves. The results showed that ZmDLR9 associates with ZmBRX1‐1, ZmBRX1‐2, and ZmGRC3, and may be involved in the processing of pre‐RNA (Figure 2d).

To assess whether the functional role of ZmDLR9 is evolutionarily conserved, we turned to the *Arabidopsis apum24‐2* mutant (SALK_033623), a previously characterized knockdown allele of the ZmDLR9 ortholog APUM24 (Maekawa et al., 2018). The *apum24‐2* exhibits reduced PR length, smaller leaves, and aberrant vein patterning (Figure S11D–H), reminiscent of the developmental defects observed in maize *dlr9‐1*. We introduced a *ZmDLR9* overexpression construct into the *apum24‐2* background and obtained two independent homozygous complementation lines (Comp‐1 and Comp‐2), as confirmed by PCR genotyping of both the transgene and the mutant allele (Figure S11A–C). Phenotypic analyses demonstrated that heterologous expression of *ZmDLR9* significantly rescued the PR length, LR number, shoot growth, and leaf venation defects of *apum24‐2* (Figure S11D–H). Thus, ZmDLR9 serves as a functional ortholog of APUM24, indicating that its role in root and shoot development is broadly conserved between maize and Arabidopsis.

Next, we analyzed pre‐rRNA processing intermediates in B73 and *dlr9‐1* via circular RT‐PCR (cRT‐PCR). Using primer pairs 18 L/25R and P24/25R, both intact 32S (A1 to B2 site) and 35S (P to B2 site) pre‐rRNA precursors were detected in 18C‐primed and 25C‐primed cDNAs. Compared with B73, the *dlr9‐1* mutant exhibited increased accumulation of both 32S and 35S pre‐rRNA (Figure 3a). Distant primer pairs (25 L/25R, P44/25R, P4/25R, and P5/25R) were used to amplify 25S rRNA, 27SB, 27SA_2/3_, and 27SA_2_ intermediates, respectively. Relative to B73, the *dlr9‐1* mutant showed elevated accumulation of 27SA_2_ and 27SB intermediates (Figure 3b), whereas no significant change was observed in intact 25S rRNA (defined by left C1 and right B2 borders) (Figure 3b). Another set of primer pairs (18 L/18R1, 18 L/18R2, 18 L/18R3, and P24/18R2) was used to amplify mature 18S rRNA (A1 to D site), 18S‐A_2_, 18S‐A_3_, and P‐A_3_ intermediates, respectively. Compared with B73, *dlr9‐1* exhibited a dramatic accumulation of the P‐A_3_ fragment, while 18S rRNA levels were comparable between B73 and the mutant (Figure 3c). Collectively, these data indicate that ZmDLR9 may be required for efficient A3 site cleavage during pre‐rRNA processing.

**Figure 3:**
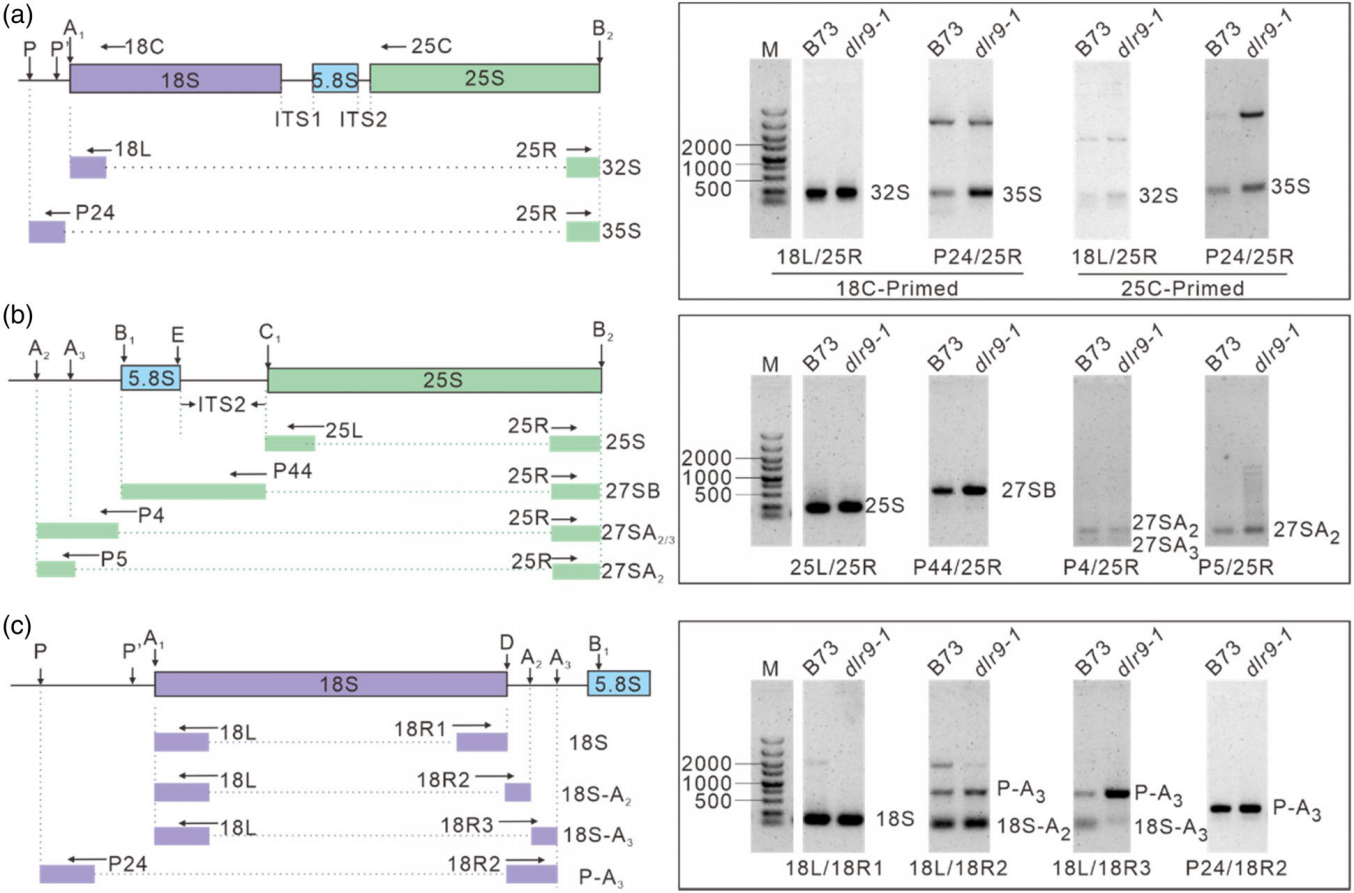
ZmDLR9 is involved in pre‐rRNA processing. (a) Mapping 5′ and 3′ ends of pre‐18S rRNAs in B73 and *dlr9‐1* mutant via cRT‐PCR with 18C‐primed cDNA. Left panel: primers for specific pre‐rRNA fragment amplification. (b) Mapping 5′ and 3′ ends of pre‐25S rRNAs in B73 and *dlr9‐1* mutant via cRT‐PCR with 25C‐primed cDNA. Left panel: primers for specific pre‐rRNA fragment amplification. (c) Mapping 5′ and 3′ ends of 35S and 32S rRNAs in B73 and *dlr9‐1* mutant via cRT‐PCR with 18C‐primed and 25C‐primed cDNA. Left panel: primers for specific pre‐rRNA fragment amplification.

### The *dlr9‐1* mutation impairs cell proliferation and triggers nucleolar stress

In Arabidopsis, the homolog of ZmDLR9, APUM24, has been shown to participate in pre‐rRNA processing by interacting with other processing factors and a putative endonuclease (Maekawa et al., 2018). To investigate whether compromised rRNA biogenesis in the *dlr9‐1* mutant causes nucleolar stress, we crossed *dlr9‐1* with stable transgenic maize lines expressing the nucleolar marker AtFIB1‐VENUS. F_1_ plants were self‐pollinated to generate an F_2_ population. Within this population, plants exhibiting the *dlr9‐1* mutant phenotype (*dlr9‐1* AtFIB1‐VENUS) were then compared with their wild‐type siblings (WT AtFIB1‐VENUS). The analysis revealed a significantly higher proportion of enlarged nucleoli in *dlr9‐1* mutant plants compared with wild‐type plants (Figure 4a,b), indicating that the *dlr9‐1* mutation disrupts normal nucleolar morphology and thereby triggers nucleolar stress.

**Figure 4:**
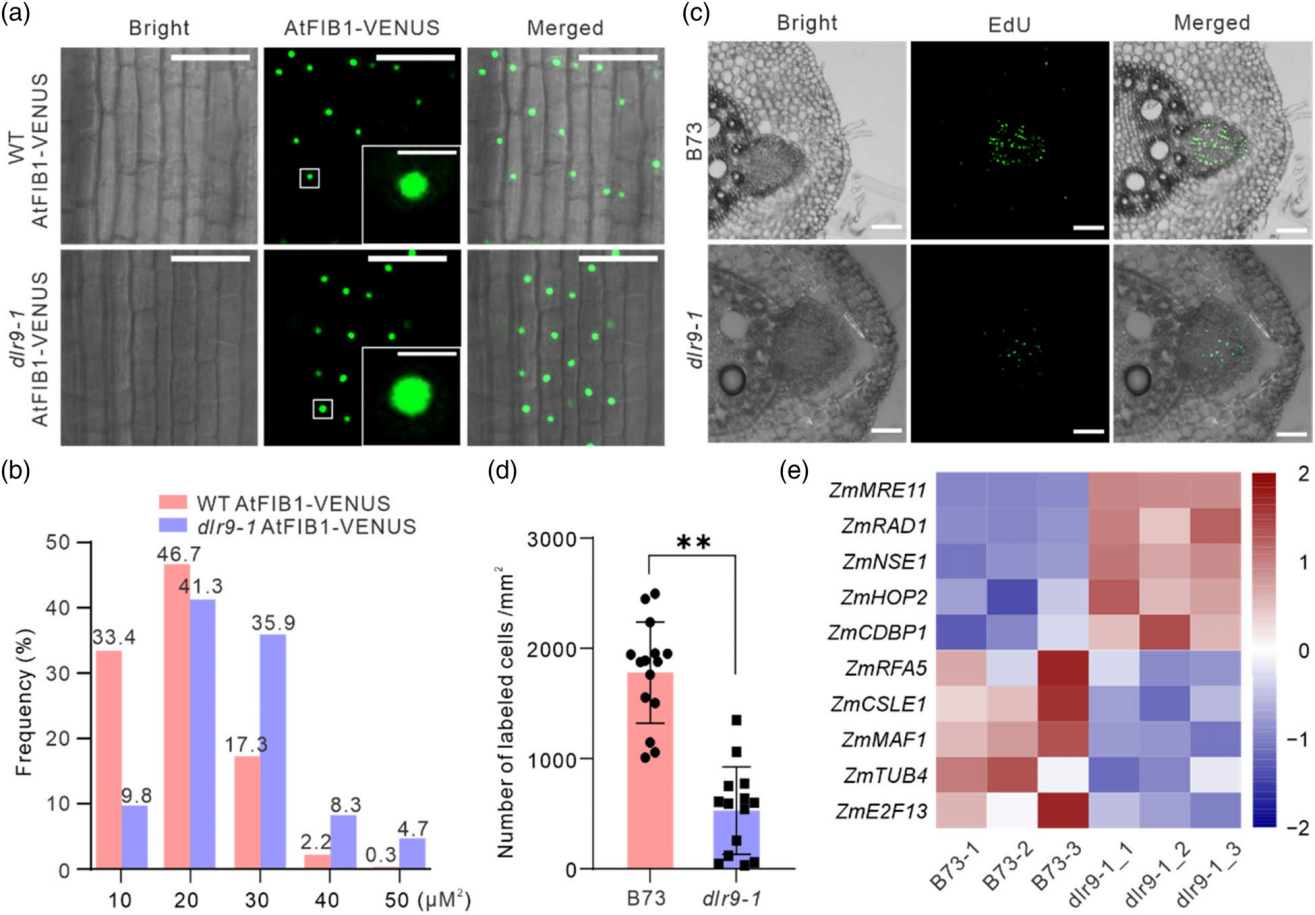
ZmDLR9 affects nucleolar size and lateral root primordia cell division activity. (a) AtFIB1–VENUS expression in WT (upper panel) and *dlr9‐1* (lower panel) merged image with differential interference contrast. Scale bar = 50 μm. (b) Nucleolar area was measured based on VENUS fluorescence intensity in 1‐week‐old roots. The histogram shows the distribution of nucleolar areas (μm^2^). *n* = 184 nucleoli. (c) Lateral root primordium cells division activity of B73 and *dlr9‐1* detected by EdU labeling. Scale bar = 100 μm. (d) Number of EdU‐labeled cells in lateral root primordium cells of B73 and *dlr9‐1*. Statistical significance was determined using Student's *t*‐test (**P < 0.01). (E) Heatmap showing normalized expression of DNA damage‐ and cell cycle‐related genes in B73 and *dlr9‐1*. Color scale represents z‐score normalized expression values; red indicates relatively higher expression, blue indicates relatively lower expression.

Given that nucleolar stress has been shown to inhibit cell proliferation in both plants and animals (Guo et al., 2023; Hua et al., 2022; Maehama et al., 2023), we next examined cell division activity in the LRP of B73 and *dlr9‐1* roots using 5‐ethynyl‐2′‐deoxyuridine (EdU) staining. Compared with the corresponding LRP cells in B73, the *dlr9‐1* exhibited a significant reduction in EdU‐positive cells (Figure 4c,d), indicating impaired cell division activity. Collectively, these transcriptomic alterations support the hypothesis that loss of ZmDLR9 function induces nucleolar stress, which may activate the DNA damage response (DDR) and disrupt cell cycle progression (Figure 4e). These molecular perturbations are likely responsible for the compromised cell proliferation and defective LRP development in *dlr9‐1*.

### ZmDLR9 is required for ribosome assembly and translation in maize

rRNAs are essential components of ribosomes, and their biogenesis abnormalities can disrupt ribosome assembly. To determine whether aberrant pre‐rRNAs in *dlr9‐1* affect this process, we performed polysome profiling on B73 and *dlr9‐1*. Ribosomes were purified using traditional biochemical methods and separated by sucrose density gradient sedimentation, followed by absorbance measurement at 260 nm. Compared with B73, *dlr9‐1* exhibited a decreased proportion of 40S and 60S ribosomal subunits and an increase in polysomes, while the 80S ribosome peak showed no obvious change (Figure 5a). These results indicate that *dlr9‐1* mutation impairs the assembly of 40S and 60S ribosomal subunits. Furthermore, we examined the abundance of ribosomal protein markers RPS6A and RPS14 (40S subunit) (Pan, Hou, et al., 2025; Wang et al., 2021) and RPL13 (60S subunit) (Le Caignec et al., 2019) in *dlr9‐1*. Compared with B73, protein levels of RPS6A, RPS14, and RPL13 were significantly reduced in *dlr9‐1* (Figure 5b), further supporting the role of ZmDLR9 in ribosome assembly in maize.

**Figure 5:**
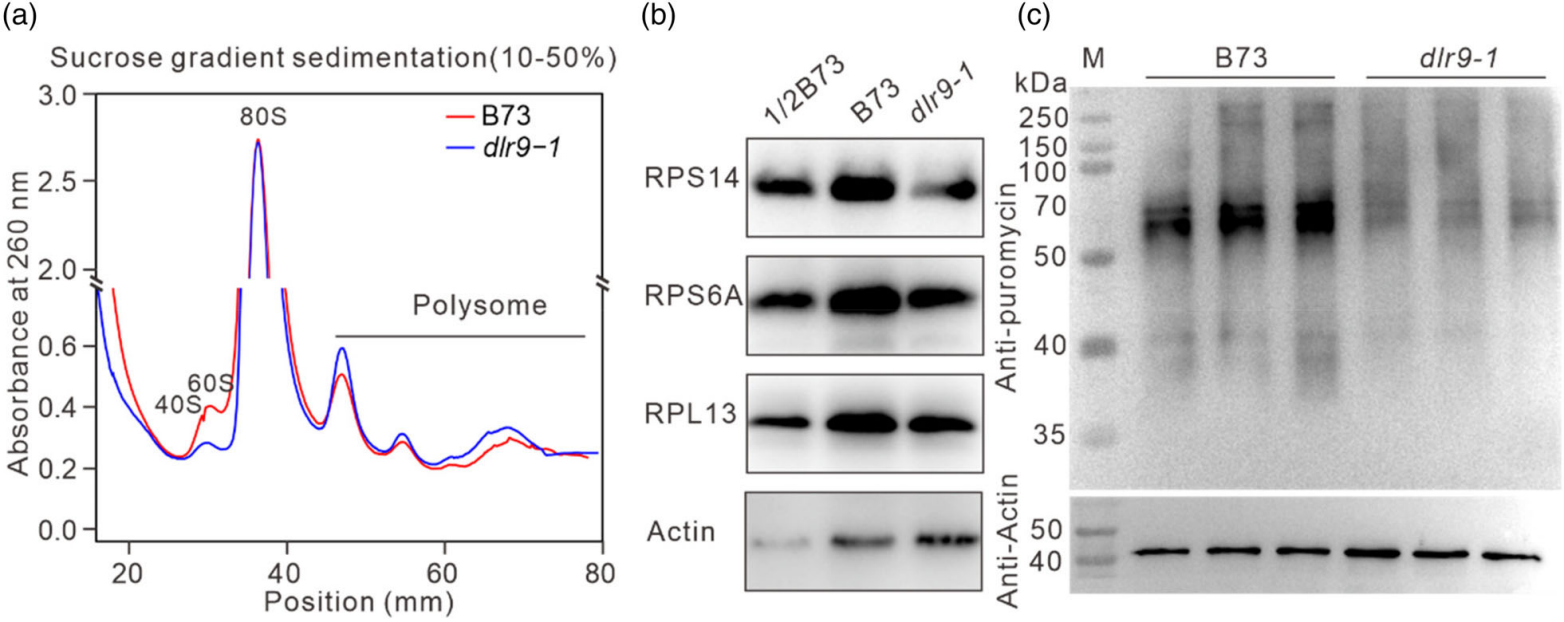
ZmDLR9 deficiency impairs ribosomal function and attenuates global translation. (a) Polysome profiles of B73 and *dlr9‐1* seedlings. Profiles were generated by monitoring absorbance at 260 nm across continuous 10–50% sucrose gradients. (b) Immunoblot analysis of RPL13, RPS6A, and RPS14 in roots of B73 and *dlr9‐1*. (c) Global translation activity assay in B73 and *dlr9‐1* seedlings (12‐day‐old). Nascent peptides labeled with puromycin were detected by immunoblotting. *Note*: Actin served as the loading control. Data are representative of three biological replicates.

Ribosomes are the central molecular machines responsible for protein synthesis (Noller, 2024; Shore & Albert, 2022). To determine whether defective ribosome assembly in the *dlr9‐1* mutant disrupts protein production, we quantified nascent polypeptide synthesis using puromycin labeling. Puromycin is an antibiotic that mimics aminoacyl‐tRNA, entering the ribosomal A‐site and covalently attaching to the growing polypeptide chain, leading to premature termination and release (Bao et al., 2020; Semenkov et al., 1992). Compared with wild‐type B73, the *dlr9‐1* mutant exhibited a pronounced reduction in global translation activity (Figure 5c), indicating that the *dlr9‐1* mutation compromises overall protein synthesis efficiency.

### ZmDLR9 is required for overall growth and development in maize

The above results demonstrate that the *dlr9‐1* mutation impairs pre‐rRNA processing, thereby disrupting ribosome biogenesis. This disruption induces nucleolar stress and compromises protein synthesis efficiency, ultimately leading to defective lateral root development. Given the fundamental role of ribosomes, we further investigated the broader impact of *dlr9‐1*. At the flowering stage, the mutant exhibited significant reductions in plant height, leaf width and length, and root area compared with B73 (Figure 6a–g). We also examined kernel‐related traits, which are critical agronomic traits in maize. The *dlr9‐1* mutant showed significantly decreased kernel width, kernel length, and 100‐kernel weight relative to B73 (Figure 6h–n). Collectively, these findings indicate that ZmDLR9 is crucial not only for lateral root formation but also for the overall growth and development of maize.

**Figure 6:**
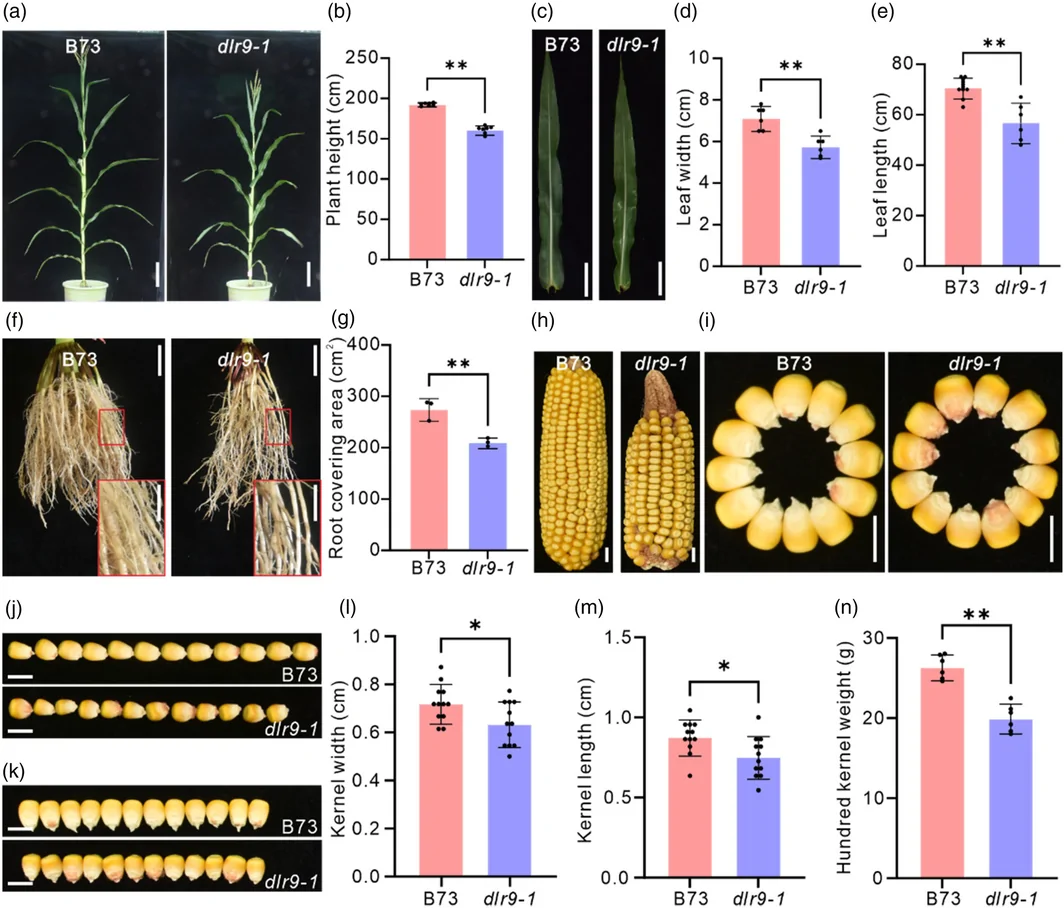
ZmDLR9 affects plant architecture and kernel‐related traits in maize. (a) Adult plant morphology of B73 and *dlr9‐1* grown in field. Scale bar = 20 cm. (b) Quantification of plant height of B73 and *dlr9‐1*. (c) Ear leaves of B73 and *dlr9‐1*. Scale bar = 10 cm. (d, e) Quantification of ear leaf width (d) and leaf length (e) of B73 and *dlr9‐1*, *n* = 6. (f) Root system architecture of B73 and *dlr9‐1*. Red boxes indicate the magnified regions shown directly below, highlighting local root traits. Scale bar = 5 cm. (g) Total root covering area of B73 and *dlr9‐1*, *n* = 3. (h–n) Ears of the B73 and *dlr9‐1* kernels at maturity (h). Phenotypes of B73 and *dlr9‐1* kernels. Kernel length of B73 and *dlr9‐1* (j, m); Kernel width of B73 and *dlr9‐1* (k, l); Scale bar = 1 cm. *n* = 12. 100‐kernel weight of B73 and *dlr9‐1* (n). Data are presented as mean ± SD. **P < 0.01, *P < 0.05 (Student's *t*‐test).

### *ZmDLR9* escaped selection during maize domestication and improvement

Genome‐wide nucleotide diversity profiles reflect the evolutionary signatures of maize domestication and intensive artificial selection. To explore the evolutionary history of *ZmDLR9*, we initially utilized the MaizeSNPDB database (http://150.109.59.144:3838/MaizeSNPDB/) to assess genome‐wide diversity patterns across distinct maize populations: modern improved varieties, traditional landraces, and the wild progenitor *Zea mays* ssp. *parviglumis*. Population genetic parameters were computed using a 10‐SNP sliding window across regions 5 kb upstream and downstream of the gene. Although the resulting nucleotide polymorphism plots appeared discontinuous, possibly due to insufficient sequencing density, the upstream and downstream regions of *ZmDLR9* exhibited similar levels of nucleotide diversity across populations, suggesting that *ZmDLR9* was not under strong selection (Figure S12A).

To further validate the above observation, we analyzed publicly available resequencing VCF data from 1515 maize accessions reported by Grzybowski et al. (2023). The dataset included 500 improved inbred lines (comprising SS, NSS, and Huangzaosi subgroups) (Table S5), 95 landraces (Table S6), and 222 teosinte accessions (including *Z. mays* ssp. *parviglumis*, *Z. mays* ssp. *mexicana*, and *Z. mays* ssp. *diploperennis*) (Table S7). Nucleotide diversity (*π*), Tajima's D, and Weir & Cockerham's *F*_*ST*_ across the *ZmDLR9* locus together with its 5 kb flanking regions (chr1: 302747203–302 763 279, B73_RefGen_V5) were estimated using a sliding window strategy. Consistent with the prior analysis, we did not observe prominent population‐specific reduction of genetic diversity within the *ZmDLR9* gene interval (light‐green shaded area, Figure S12B). Collectively, these population genetic results suggest that *ZmDLR9* has not undergone strong artificial selective sweep during maize domestication and improvement.

## DISCUSSION

Pumilio RNA‐binding proteins are crucial post‐transcriptional regulators across eukaryotes (Huh et al., 2013; Sonoda & Wharton, 2001). Previous studies in Arabidopsis have shown that several plant PUM proteins are involved in diverse biological processes, including viral defense, leaf morphogenesis, embryogenesis, seed development, and adaptation to abiotic stress (Abbasi et al., 2010; Huang et al., 2021; Shanmugam et al., 2017). Notably, their roles in root development have remained unexplored. Here, we identify a maize PUM protein, ZmDLR9, that is involved in lateral root development, thereby expanding the functional repertoire of plant PUF proteins.

### ZmDLR9 is a conserved yet functionally diversified nucleolar PUF protein

Phylogenetic analysis places ZmDLR9 within Clade II of the PUM family, closely related to Arabidopsis APUM23 and APUM24, both nucleolar proteins involved in pre‐rRNA processing (Abbasi et al., 2010; Shanmugam et al., 2017). Consistent with this evolutionary relationship, ZmDLR9 localized specifically to the nucleolus in both tobacco and maize cells (Figure 2b,c), and its heterologous expression rescued the root, shoot, and leaf venation defects of the *Arabidopsis apum24‐2* mutant (Figure S11). This confirms a deep functional conservation in core nucleolar activities. However, significant functional divergence is evident. While the *Arabidopsis apum24‐2* mutant shows defects in both primary and lateral roots, the maize *dlr9‐1* mutant specifically impairs lateral root development without affecting PR elongation (Figure 1a; Figure S1; Figure S11). Although the mechanistic basis for this phenotypic discrepancy remains to be fully elucidated, it is possible that lateral root primordia (LRP), which rely on vigorous and temporally coordinated cell divisions for their formation and emergence, are particularly vulnerable to perturbations in ribosome biogenesis. Variation in spatiotemporal gene expression patterns or genetic redundancy may also contribute to this difference. Further mechanistic work will be required to dissect the underlying causes. This suggests lineage‐specific adaptations in how this conserved module regulates root system architecture. Furthermore, phenotypic divergence extends to nucleolar morphology: *apum24* mutants exhibit nucleolar shrinkage under stress (Maekawa et al., 2018), whereas *dlr9‐1* mutants display an increased frequency of enlarged nucleoli (Figure 4a,b), a phenotype reminiscent of *Arabidopsis apum23* mutants (Abbasi et al., 2010). Although ZmDLR9, APUM23, and APUM24 all participate in pre‐rRNA processing, they likely function at distinct steps or within different ribonucleoprotein complexes, which may account for the differential nucleolar phenotypes. This indicates that within this closely related clade, phylogenetic position does not strictly predict phenotypic outcome, highlighting an evolutionary trajectory where conserved molecular function (rRNA processing) can be integrated into distinct developmental and stress response circuits in different species.

### A mechanistic cascade from rRNA processing to lateral root development

Impaired rRNA biogenesis induces nucleolar stress, which is typically characterized by aberrant nucleolar morphology such as enlargement or reduced size (Holmberg Olausson et al., 2012; Zhou et al., 2015). In most instances, nucleolar enlargement has been observed to coincide with the excessive accumulation of intermediates generated during pre‐rRNA processing (Ohbayashi & Sugiyama, 2018; Sáez‐Vásquez & Delseny, 2019). In HGPS (Hutchinson–Gilford syndrome) cells, hypomethylation of the rDNA promoter allows RNA polymerase I to engage more templates, resulting in enlarged nucleoli, increased pre‐rRNA synthesis, and consequently elevated levels of mature 28S and 18S rRNA (Phan et al., 2019). Cytological studies have revealed that the nucleolus is enlarged in mutants such as *tormoz* (Griffith et al., 2007), *nof1* (Harscoët et al., 2010), *apum23* (Abbasi et al., 2010), *rid2* (Ohbayashi et al., 2011; Shinohara et al., 2014), *ren1*(Reňák et al., 2014), *la1* (Fleurdepine et al., 2007), and *domino1* (Lahmy et al., 2004) relative to wild‐type plants. By contrast, the nucleolus is smaller in *fem111* mutants (Portereiko et al., 2006) and displays a disorganized structure in *Atnuc‐L1* plants (Pontvianne et al., 2007), whereas *apum24* nucleoli become smaller in the presence of 50 mM Glc (Maekawa et al., 2018). Mechanistically, delayed processing of the small subunit pre‐rRNA activates the nucleolar quality control system, reducing rRNA outward diffusion and subsequently leading to structural reorganization of the nucleolus—often resulting in expansion or even rupture of its inner region (Pan, Shan, et al., 2025).

Our data delineate a clear pathway from molecular function to developmental phenotype. We show that *dlr9‐1* mutants accumulate multiple pre‐rRNA processing intermediates (35S, 32S, 27SA2, 27SB), indicative of impaired ribosome biogenesis (Figure 3a–c). This defect has direct cellular consequences: (1) disrupted assembly of 40S and 60S ribosomal subunits, as shown by polysome profiling and reduced abundance of ribosomal proteins (Figure 5a,b); (2) severely compromised global protein synthesis, evidenced by nearly absent puromycin‐labeled nascent polypeptides (Figure 5c); and (3) nucleolar stress, manifested as aberrant nucleolar enlargement (Figure 4a,b). The high expression of *ZmDLR9* in meristematic tissues like young roots aligns with the particular vulnerability of these regions. Eukaryotic ribosome assembly is a highly orchestrated process that involves over 200 protein factors (Lau et al., 2023; Zhang et al., 2023). LRP are sites of intense cell division and growth, processes with exceptionally high demand for ribosomes and protein synthesis. The EdU staining revealed reduced cell division activity specifically within the LRP of *dlr9‐1*, explaining why primordia initiation is normal but their subsequent development is arrested. Thus, ZmDLR9 deficiency creates a bottleneck in translational capacity. This insufficiency is most harmful in rapidly proliferating tissues. Consequently, lateral root outgrowth is specifically suppressed.

Beyond the translational limitation caused by defective ribosome biogenesis, our transcriptomic profiling further revealed potential associations between nucleolar stress and altered cell cycle checkpoint activity in *dlr9‐1*. Transcriptomic analysis detected elevated transcript levels of core DDR genes, including *ZmMRE11*, *ZmNSE1*, and *ZmHOP2*, accompanied by perturbed expression of cell cycle regulatory genes. Specifically, the cell proliferation activator *ZmE2F13* was downregulated, while the G1/S transition suppressor *ZmCDBP1* was upregulated, which together may reflect moderate activation of DNA damage checkpoint signaling. Considering the essential role of ZmDLR9 in pre‐rRNA processing and ribosome maturation (Figure 3), it is plausible that defective rRNA biogenesis and subsequent nucleolar stress contribute to the observed molecular perturbations in *dlr9‐1*. In rapidly dividing lateral root primordium cells, sustained nucleolar stress may potentially provoke transcription–replication conflicts and abnormal R‐loop accumulation, which are common endogenous stimuli for DDRs and checkpoint activation. Such cell cycle checkpoint perturbation could at least partially explain the reduced cell proliferation in *dlr9‐1* LRP, consistent with our EdU staining results. Collectively, these findings suggest a tentative regulatory cascade, in which loss of *ZmDLR9* function disrupts rRNA processing and induces nucleolar stress, which may trigger DNA damage‐associated checkpoint responses, compromise cell division activity, and ultimately contribute to the defective lateral root development of maize *dlr9‐1*. Nevertheless, further functional validation is still required to confirm the direct causal relationship between these transcriptomic perturbations and the impaired cell proliferation phenotype.

### Evolutionary stability and agricultural implications

While both natural and artificial selection have profoundly shaped the genomes and phenotypes of crops (Xu et al., 2024), the pre‐rRNA processing gene *ZmDLR9* appears to have escaped the pronounced selective sweeps typically associated with domestication and crop improvement. Population genetic analyses integrating nucleotide diversity (*π*), Tajima's D, and Weir‐Cockerham *F*_*ST*_ revealed no consistent genome‐wide signatures of strong artificial positive selection at the *ZmDLR9* locus across teosinte, landraces, and modern maize inbred lines (Figure S12). This pattern is characteristic of genes encoding core cellular machinery, where functional constraint limits permissible variation and purifying selection maintains sequence integrity (Zhang & Li, 2004). This evolutionary stability stands in sharp contrast to the dynamic selection histories seen in regulatory genes controlling discrete morphological traits such as *tb1* (Studer et al., 2011), suggesting that *ZmDLR9* represents a “buffered” genetic node whose optimal function is non‐negotiable for cellular homeostasis.

However, the significant reduction in kernel size and weight in the *dlr9‐1* mutant demonstrates that its function directly impacts yield‐related traits. The mutant phenotypes mirror those of other maize ribosome biogenesis mutants (*urb2*, *shrek1*), reinforcing the notion that ribosomal homeostasis is a key determinant of plant growth and productivity (Liu et al., 2022; Wang et al., 2018). Loss‐of‐function *urb2* mutants display pronounced accumulation of pre‐rRNA intermediates (35/33S rRNA, P‐A3, and 18S‐A3), ultimately resulting in defective kernel development (Wang et al., 2018). Similarly, disruption of SHREK1, which is required for pre‐60S ribosomal particle assembly, leads to embryo abortion, insufficient endosperm development, and complete embryonic lethality (Liu et al., 2022). These collective findings reinforce the notion that ribosomal homeostasis represents a non‐redundant node governing plant growth and productivity (Weis et al., 2015). The *dlr9‐1* displays widespread pleiotropic defects, including reduced plant height, smaller leaf size, diminished total root area, shorter kernels, and decreased 100‐kernel weight across shoots, roots, and kernels. These comprehensive developmental abnormalities demonstrate that ZmDLR9 exerts widespread regulatory functions throughout maize development, rather than acting exclusively in lateral root specification. This organ‐wide functional requirement is consistent with the essential housekeeping role of ribosome biogenesis in plant growth. A plausible mechanistic interpretation for this organ‐specific phenotypic variation is that tissues with high cell proliferation activity, including nascent LRP, are hypersensitive to disruptions in ribosome biogenesis and function. This finding further suggests that post‐embryonic root branching can serve as a sensitive and reliable in vivo indicator for evaluating the homeostasis of ribosome biogenesis in maize. The functional conservation of this pathway extends beyond maize. In *Arabidopsis thaliana*, the null allele of *APUM24* (the ortholog of *ZmDLR9*) causes embryonic lethality due to defects in both gametogenesis and embryogenesis (Maekawa et al., 2018), highlighting the indispensable nature of this PUF‐domain protein family throughout the plant life cycle. While *ZmDLR9* does not appear to have been a direct target of historical selection during maize domestication, its integrity remains foundational for agricultural performance. The tight functional constraint observed at this locus implies that conventional allele mining approaches may yield limited natural variation for breeding applications. Core biogenesis factors exert a surprisingly strong influence on plant phenotypes, even when their activity or expression is only mildly altered. This implies that precisely regulating these factors could open up new strategies for fine‐tuning plant growth architecture. Such precise modulation might be achieved through various approaches, including the use of tissue‐specific promoters, developmental stage‐specific knockdowns, or conditional expression systems.

## CONCLUSION

In summary, we have identified *ZmDLR9* as a crucial regulator of lateral root development in maize. Its function extends beyond typical RNA target binding to a central role in nucleolar rRNA processing, thereby ensuring efficient ribosome assembly and protein translation. This ribosome biogenesis pathway is particularly critical for sustaining the high metabolic demands of developing LRP. Our study bridges a gap in PUF protein biology, linking a specific nucleolar PUF member to the regulation of root system architecture through a fundamental cellular mechanism. It also highlights how conserved cellular machinery can be tailored to achieve distinct developmental outcomes in different plant lineages and underscores the importance of ribosomal homeostasis as a cornerstone of plant growth and potential agricultural yield.

## MATERIALS AND METHODS

### Plant materials and growth conditions

*dlr9‐1* allele was derived from an EMS‐mutagenized population of maize inbred line B73, while *dlr9‐2* allele was acquired from Maize EMS‐Induced Mutant Database (maizeEMSDB) (Lu et al., 2018). To genetically map the target gene, *dlr9‐1* mutant was crossed with wild‐type B73 to generate F_1_ progeny. F_1_ plants were then self‐pollinated to produce an F_2_ segregating population. Both F_1_ and F_2_ generations were cultivated in experimental fields in Hainan, China. Uniformly sized maize seeds were selected for phenotypic screening and lateral root analysis. Seeds were washed with ddH_2_O, soaked overnight, and germinated using rolled towel method on moistened filter paper at 28°C. For subcellular localization, etiolated leaves from 1‐week‐old B73 maize seedlings cultivated in the darkness at 28°C were used for protoplast isolation. *N. benthamiana* seedlings were planted at 22°C under a 16‐h light/8‐h dark photoperiod.

Field experiments were conducted in Hainan, China, during the winter growing season of 2022. Plants were grown under standard field management practices with a row spacing of 60 cm and a plant spacing of approximately 25 cm. Both the *dlr9* and B73 were planted in three replicate rows, with each row measuring 4 m in length. At physiological maturity, all ears were harvested for trait evaluation. Kernel‐related traits, including 100‐kernel weight, kernel length, and kernel width, were measured from air‐dried seeds.

*Arabidopsis thaliana* Columbia‐0 (Col‐0) ecotype served as the wild‐type control. *apum24‐2* mutant (SALK_033623) was obtained from the Nottingham Arabidopsis Stock Centre (NASC). Col‐0 and mutants were grown on soil under a 16‐h light/8‐h dark photoperiod at 22°C. For phenotypic analysis, Col‐0, *apum24‐2*, and complementation lines (Comp‐1, Comp‐2) were surface‐sterilized with 10% sodium hypochlorite, stratified at 4°C for 3 days, and germinated on MS medium. Genotyping of *apum24‐2* and Comp‐1, Comp‐2 was performed by PCR using the following primer combinations: T‐DNA insertion verification (F1/R1 and F1/R2), complementation lines (*ZmDLR9* gene‐specific amplification: R3 and UBQ promoter: F2). All primer sequences are listed in Table S8.

### Generation of transgenic Arabidopsis plants

For complementation assay, *ZmDLR9* was amplified from B73 cDNA and cloned into pDONR207 vector to generate pDONR207‐ZmDLR9. The gene was subsequently transferred into the pCUN_GW_VENUS destination vector via LR recombination. Transgenic plants were generated by transforming *apum24‐2* plants via *Agrobacterium tumefaciens* GV3101‐mediated floral dip. T_1_ seedlings were selected on MS medium containing basta (10 mg/L) and chloramphenicol (25 mg/L). T_2_ seeds were germinated on the same selective medium, and segregation ratios were quantified. Putative complementation lines were verified by PCR genotyping using primers listed in Table S8.

### EdU staining

Taproot segments (1 cm in length) were excised from 4‐day‐old seedlings of B73 and *dlr9‐1* mutant maize line, previously cultured in Hoagland nutrient solution. After blotting dry with absorbent paper, segments were incubated in 300 μL of 10 μM EdU reagent (Cell‐Light EdU Apollo488 In Vitro Kit, Ribobio) for 10 h in darkness at room temperature to label proliferating cells. Following labeling, segments underwent sequential processing: two washes in PBS buffer (5 min each), fixation in 4% (w/v) paraformaldehyde (PFA) in PBS for 30 min at room temperature, and quenching of residual PFA with 500 μL of 2 mg/mL glycine in PBS for 5 min. After an additional PBS wash (5 min), tissues were permeabilized with 0.5% (v/v) Triton X‐100 in PBS for 10–20 min. Samples were then embedded in 4% (w/v) low‐melting point agarose and sectioned using a vibratome. Tissue sections were subsequently stained by incubation with 100 μL of 1 × Apollo reaction mixture for 30 min at room temperature in the dark. Unbound stain was removed by washing twice with 500 μL of kit's permeabilization wash solution (10 min per wash), followed by a brief rinse in PBS. Finally, EdU‐labeled sections were imaged using confocal laser scanning microscopy.

### Feulgen staining

Root segments were excised from PRs of 4‐day‐old seedlings of B73 and *dlr9‐1*. After blotting dry with absorbent paper to remove surface moisture, segments were placed in a clean Petri dish. Tissue samples were then completely submerged in freshly prepared 6 N HCl and subjected to vacuum infiltration for 30 min to facilitate acid hydrolysis. Following hydrolysis, the HCl solution was carefully removed by aspiration. Samples were subsequently rinsed 3–5 times with ddH_2_O to remove residual acid. For nuclear staining, segments were immersed in Feulgen stain (Schiff's fuchsin‐sulfite reagent, Sigma‐Aldrich, St. Louis, MO, USA) at room temperature for 5–10 min. Finally, the stained root segments were imaged using a flatbed scanner (Epson Expression 11000XL) to visualize and record the distribution of nuclear DNA.

### Paraffin section preparation and observation

The PRs of maize B73 and *dlr9‐1* seedlings germinated on filter paper for 4 days were collected and fixed in 50% FAA fixative solution. The samples were then processed through gradient ethanol dehydration, xylene transparency, and paraffin embedding. Continuous thin sections were prepared using a Leica paraffin microtome. The fresh sections were directly observed and photographed under a light microscope without dewaxing, rehydration, and staining for phenotypic recording and statistical analysis.

### RNA isolation and RNA‐seq analsysis

Seeds of maize inbred line B73 and *dlr9‐1* were germinated. Uniform seedlings were hydroponically cultured in Hoagland's nutrient solution for 4 days after 2 days of germination. Root tissues were harvested, frozen in liquid nitrogen immediately, and stored at −80°C. Total RNA was extracted using the RNAsimple Total RNA Kit (Cat. No. DP419, TIANGEN Biotech (Beijing) Co., Ltd., China), and qualified RNA samples from three biological replicates per genotype were used for transcriptome library construction and Illumina sequencing. Raw sequencing reads were filtered using fastp to obtain high‐quality clean reads, which were then aligned to the maize B73 RefGen_v4 reference genome. Differential expression analysis was performed using the R package DESeq2. Genes with an absolute fold change ≥1.5 and an adjusted *P*‐value < 0.05 were defined as differentially expressed.

### Phylogenetic analysis

The protein sequence of ZmDLR9 was used as the query to identify its homologs in the *Arabidopsis thaliana* genome database (TAIR; https://www.arabidopsis.org/), MaizeGDB (https://maizegdb.org/), and the NCBI non‐redundant protein database. Multiple sequence alignment of ZmDLR9 and its homologs was performed using ClustalW. The protein sequences were subsequently visualized using GeneDoc, with conservation levels color‐coded according to the method described by Zhang et al. (2026). A phylogenetic tree was constructed with MEGA11 software using the neighbor‐joining method and bootstrap analysis with 1000 replicates.

### Subcellular localization analysis

To determine subcellular localization of ZmDLR9, open reading frame (ORF) of ZmDLR9 without a stop codon was cloned into binary vector pK7RWG2 to generate pK7RWG2‐ZmDLR9‐RFP construct. For co‐localization analysis, pK7FWG2‐AtFIB1‐GFP vector (serving as a nucleolus marker) was also prepared. Resulting plasmids were introduced into *Agrobacterium tumefaciens strain* GV3101. Transient expression assays were performed using maize protoplasts isolated from etiolated 1‐week‐old maize seedling leaves, following the polyethylene glycol (PEG)‐mediated transformation method (Cao et al., 2014). Similar subcellular localization analyses have been previously described for other maize proteins (Li et al., 2025). Protoplasts were co‐transfected with pK7RWG2‐ZmDLR9‐RFP and pK7FWG2‐AtFIB1‐GFP to visualize the subcellular distribution of ZmDLR9‐RFP. Fluorescence signals were monitored using a ZEISS LSM 980 confocal laser scanning microscope. ZmDLR9‐RFP was excited at 561 nm (16% laser power) and detected at 570–641 nm, while AtFIB1‐GFP was excited at 488 nm (6.5% laser power) and detected at 490–552 nm. For *N. benthamiana* leaves, ZmDLR9‐RFP was excited at 561 nm (2.4% laser power) and detected at 570–694 nm, and AtFIB1‐GFP was excited at 488 nm (0.09% laser power) with detection at 490–579 nm. Fluorescence signals were visualized using an LSM980 confocal microscope (ZEISS, Oberkochen, Germany).

### Bimolecular fluorescence complementation (BiFC) assays

To investigate potential protein–protein interactions, BiFC assays were performed. The coding sequence of ZmDLR9 was fused to the N‐terminal fragment of yellow fluorescent protein (nYFP) to generate ZmDLR9‐YN. Putative maize orthologs of Arabidopsis AtBRX1‐1, AtBRX1‐2, and AtGRC3 – designated ZmBRX1‐1, ZmBRX1‐2, and ZmGRC3 – were individually fused to the C‐terminal fragment of YFP (cYFP), resulting in ZmBRX1‐1‐YC, ZmBRX1‐2‐YC, and ZmGRC3‐YC constructs. As negative controls, GUS was fused to both nYFP and cYFP (GUS‐YN and GUS‐YC). All constructs were co‐expressed with a nucleolar marker, mCherry‐AtFIB1, in 4‐week‐old *Nicotiana benthamiana* leaves. After 48 h of incubation at 22°C, reconstituted YFP signals were visualized using a ZEISS LSM980 confocal microscope. YFP was excited at 514 nm (15% laser power) and detected at 508–579 nm, while mCherry was excited at 561 nm (12% laser power) and detected at 570–658 nm.

### cRT‐PCR analysis

The 5′ and 3′ ends of various rRNA precursors were determined by circular RT‐PCR (cRT‐PCR) as previously described (Perrin et al., 2004) with minor modifications. Briefly, 10 μg of total RNA isolated from coleoptiles of both B73 and *dlr9‐1* was denatured at 70°C for 5 min and immediately chilled on ice for 2 min. The RNA was then self‐ligated in a 50 μL reaction containing 30 U of T4 RNA ligase 1 (New England Biolabs), 1× T4 RNA ligase buffer, 1 mM ATP, and 40 U of RNase inhibitor. The ligation reaction was incubated at 37°C for 2 h, followed by heat inactivation at 65°C for 15 min. Circularized RNA was precipitated by adding 5 μL of 3 M sodium acetate (pH 5.2), 2 μL of glycogen (20 mg/mL), and 171 μL of 100% ethanol, and incubated at −20°C overnight. After centrifugation at 15 300 *×* ***g*** for 15 min at 4°C, the pellet was washed with 70% ethanol, air‐dried, and resuspended in DEPC‐treated water. First‐strand cDNA was synthesized from the circularized RNA using the PrimeScript™ RT Reagent Kit (Takara Biomedicals, Otsu, Japan) with gene‐specific primers. Primer sequences used for amplification of 32S, 27S, 18S, and the P‐A3 intermediate are listed in Table S8.

### RT‐qPCR analysis

Total RNA was extracted from various tissues of B73 maize plants, including roots, stems, and leaves of seedlings, as well as roots, stems, leaves, embryos, silks, tassels, and ears of adult plants. TransZol reagent (Thermo Fisher Scientific, Waltham, MA, USA) was used for RNA extraction. For RT‐qPCR, first‐strand cDNA was synthesized from 1 μg of total RNA using the PrimeScript™ RT Reagent Kit (Takara Biomedical Technology Co., Ltd., Dalian, China). All RT‐qPCR analyses were performed on a Roche LightCycler480 System using LightCycler SYBR Green I Master kit (Roche). *UBQ10* gene was used as an internal control to normalize expression levels. Relative quantification of mRNA was calculated using comparative Ct (2^–ΔΔCt) method. Three biological replicates were performed for each experiment. All primer sequences are listed in Table S8.

### Microscopy of leaf shape

Cotyledons and true leaves were excised from 3‐week‐old *Arabidopsis thaliana* plants grown under greenhouse conditions. Tissues were decolorized by immersion in 1.5 mL of 95% ethanol for 1 h at room temperature. After ethanol removal, samples were cleared in 1 mL of chloral hydrate solution with continuous rotation on an orbital shaker for 5–6 h until fully transparent. Cleared tissues were subsequently washed by transferring to a six‐well plate containing 3 mL of ddH_2_O per well. Leaf samples were then mounted on glass slides and gently compressed under coverslips. Vein patterning was documented using brightfield imaging on a stereomicroscope (Axio Zoom.V16) equipped with a macro zoom objective and a digital camera system.

### Measurement of nucleolar size

To measure nucleolar size, transgenic lines expressing nucleolar marker AtFIB1‐VENUS were crossed with *dlr9‐1* to generate F_1_ progeny. F_1_ plants were self‐pollinated to produce an F_2_ population. From F_2_ generation, homozygous *dlr9‐1* mutant lines carrying AtFIB1‐VENUS transgene (designated as *dlr9‐1* AtFIB1‐VENUS) were isolated, along with homozygous wild‐type background lines expressing AtFIB1‐VENUS (WT AtFIB1‐VENUS), which served as controls. Seeds of both WT AtFIB1‐VENUS and *dlr9‐1* AtFIB1‐VENUS were germinated on filter paper and grown for 4 days. Vibratome sections were then prepared from seedlings, and nucleoli were visualized via GFP fluorescence using confocal laser scanning microscopy. Sizes of nucleoli in GFP‐positive cells were measured and quantified using ImageJ software. VENUS fluorescence signals were observed under an LSM980 (ZEISS) confocal microscope. Excitation was performed at 514 nm (3% laser power), and emission was collected at 517–561 nm with a pinhole set to 1.03 Airy units (29 μm).

### Polysome profiling and sucrose gradient fractionation

Polysome profiling was performed as previously described (Song et al., 2024) with minor modifications. Approximately, 0.5 g of frozen seedling tissue was ground into a fine powder in liquid nitrogen. The powder was thoroughly mixed with 1 mL of polysome extraction buffer (PEB) containing 0.2 M Tris–HCl (pH 8.0), 0.2 M KCl, 35 mM MgCl_2_, 25 mM EGTA, 1% sodium deoxycholate, 2% polyoxyethylene‐10‐tridecyl ether (PTE), 100 mM 2‐mercaptoethanol, 50 μg/mL cycloheximide (CHX), and 50 μg/mL chloramphenicol (CHL). After incubation on ice for 30 min, cell debris was removed by centrifugation at 21 130 *×* ***g*** for 30 min at 4°C. The RNA concentration of the supernatant was measured, and an amount equivalent to an OD_260_ value greater than 5000 was loaded onto a 10%–50% sucrose gradient. Gradients were ultracentrifuged at 95329 *×* ***g*** for 4 h at 4°C using a Beckman SW‐40Ti rotor. Polysome profiles were monitored by continuous measurement of absorbance at 260 nm using a Piston Gradient Fractionator (BioComp Instruments) coupled with a Model EM‐1 Econo UV Monitor (Bio‐Rad, Hercules, CA, USA).

### Puromycin labeling assay

Nine‐day‐old B73 and *dlr9‐1* mutant seedlings grown in liquid Hoagland nutrient solution were transferred to 50 mL of fresh solution containing 10 μg/mL puromycin (Invitrogen) for translation inhibition. Samples were subjected to three additional rounds of vacuum infiltration, each lasting 1 min, with 5‐min intervals under a reduced pressure of −80 kPa (gauge reading −0.8 on the 100 × kPa scale). Samples were collected 30 min after third infiltration, flash‐frozen in liquid nitrogen, and subsequently used for protein extraction and immunoblotting. Total protein extracted from an equal amount of each sample was separated by SDS‐PAGE, transferred electrophoretically to a PVDF membrane, and immunoblotted with an anti‐puromycin antibody (Merck, Darmstadt, Germany). Anti‐ACTIN was used as a loading control.

### Population genetic analyses

Population genetic evaluation of the *ZmDLR9* locus was performed using two independent SNP datasets. First, SNP data were retrieved from the MaizeSNPDB database (http://150.109.59.144:3838/MaizeSNPDB/; Zhou et al., 2019), which is based on the B73_RefGen_V4 reference genome, to estimate nucleotide diversity across modern improved varieties, traditional landraces, and *Zea mays* ssp. *parviglumis*. Population genetic statistics were calculated with a 10‐SNP sliding window covering the *ZmDLR9* gene and its 5 kb upstream and downstream flanking regions (Figure S12A).

For further validation, we utilized publicly available resequencing VCF data from Grzybowski et al. (2023). A total of 817 maize accessions with clear population classification were retained for population genetic analyses, including 500 improved inbred lines belonging to the SS, NSS, and Huangzaosi heterotic groups (Table S5), 95 traditional landraces (Table S6), and 222 wild teosinte accessions representing diverse teosinte germplasms and wild *Zea taxa* (Table S7). Accessions with ambiguous population affiliation were excluded to ensure the accuracy of group‐based statistical comparisons. Nucleotide diversity (*π*), Tajima's D, and Weir & Cockerham's *F*_*ST*_ were computed across the target interval chr1: 302747203–302 763 279 (B73_RefGen_V5 reference genome), which spans the *ZmDLR9* locus (chr1: 302752203–302 758 279) plus 5 kb flanking sequences. For this dataset, statistics were calculated using a 1‐kb physical distance sliding window. Nucleotide diversity was used to quantify within‐population genetic variation. Tajima's D was applied to test deviations from neutral evolutionary expectations. Pairwise *F*_*ST*_ was calculated to assess genetic differentiation among teosinte, landrace, and improved maize groups. All population genetic outputs were visualized to characterize variation patterns around *ZmDLR9* (Figure S12B).

## AUTHOR CONTRIBUTIONS

ZH and DL designed all experiments, analyzed the data, and wrote the manuscript. CW, YL, AX, and SZ performed the experiments and prepared the data; YZ, FW, HZ, TZ, and ZH edited and revised the manuscript. All authors read and approved the final manuscript.

## CONFLICT OF INTEREST

All authors declare that there are no conflicts of interest.

## Supporting information


**Figure S1.** Phenotypic identification of *dlr9‐1*.
**Figure S2.** Morphological characterization and quantitative analysis of lateral root primordium (LRP) development in B73 and *dlr9‐1*.
**Figure S3.** Genetic segregation of root phenotypes in *dlr9‐1*.
**Figure S4.** Whole‐genome and chromosome 1 SNP index plots from MutMap analysis of *dlr9‐1*.
**Figure S5.** The 500 bp upstream and downstream sequences flanking the mutation site of *Zm00001d034750* in *dlr9‐2*.
**Figure S6.** Alternative splicing of *ZmDLR9* gene in *dlr9‐2*.
**Figure S7.** Genetic allelism test and root phenotypes of *dlr9* alleles.
**Figure S8.** Phylogenetic analysis of APUM protein family in maize and Arabidopsis.
**Figure S9.** Sequence alignment and conservation domain analysis of ZmDLR9.
**Figure S10.** Analysis of *ZmDLR9* transcripts.
**Figure S11.** Heterologous expression and functional validation of ZmDLR9 in Arabidopsis.
**Figure S12.** Population nucleotide diversity analysis of *ZmDLR9* in maize.

**Table S1.** The lateral root of F_2_ population evaluated.
**Table S2.** Mapping of the *dlr9‐1* locus to a 6.09 Mb interval (positions 300 944 916–307 039 376 bp) near the telomere of chromosome 1.
**Table S3.** Variant sites within the candidate interval with SNP index values.
**Table S4.** High‐frequency EMS‐induced SNPs (G → A/C → T, Frequency >0.8).
**Table S5.** Subgroup classification of 500 maize inbred lines (SS, NSS, Huangzaosi) from Grzybowski et al. (2023) Supporting Information.
**Table S6.** Subgroup classification of 95 maize landraces lines from Grzybowski et al. (2023) Supporting Information.
**Table S7.** Subgroup classification of 222 teosinte accessions from Grzybowski et al. (2023) Supporting Information.
**Table S8.** Primers used in this study.

## Data Availability

Sequence data are available under the following accession numbers: *ZmDLR9* (*Zm00001d034750*), *dlr9‐2* (*EMS4‐0 dc597*), *APUM24* (*At3g16810*), *ZmBRX1‐1* (*Zm00001d050923*), *ZmBRX1‐2* (*Zm00001d017049*), and *ZmGRC3* (*Zm00001d039568*). Raw sequencing reads were deposited in the Genome Sequence Archive (GSA) under CRA049060 (ftp://download.big.ac.cn/gsa5/CRA049060). The data supporting the findings of this study are available within the article and its Supplementary Materials.
