# Supplementary material for: ZmDLR9 is required for lateral root development via pre‐rRNA processing in maize
Source: Plant J. 2026 Oct 8;128(1):e71156. doi: 10.1111/tpj.71156 (PMC13646893; doi:10.1111/tpj.71156)
Supplement: Supplementary file 1 [file TPJ-128-0-s001.docx]

**Figure S1.** Phenotypic identification of *dlr9-1*. (A-B) Time-course statistical analysis of lateral root number (A-B) and primary root length (B) in B73 and *dlr9-1* seedlings. Data are presented as mean ± SD, n = 5. (C-D) Comparison of primary root length (C) and number of lateral roots (D) in the B73 and *dlr9-1* seedlings at 7 days. (E) Lateral root primordia, as revealed by Feulgen staining, in 4-day-old B73 and *dlr9-1* seedlings of germination. The red arrow indicates a lateral root primordium. Scale bar = 1 cm. (F) Number of lateral root primordia in the primary root of 4-day-old B73 and *dlr9-1* seedlings. Error bars represent the standard deviation (SD) from three independent roots. Statistical significance was determined using Student's *t*‐test, ***p* < 0.01, ns: not significant compared with B73.

**Figure S2**. Morphological characterization and quantitative analysis of lateral root primordium (LRP) development in B73 and *dlr9-1*. (A) The lateral root primordium developmental stages (S1–S6) in maize roots. S1: pericycle cell initiation; S2: primordium protrusion toward the cortex; S3: primordium length exceeding 2/5 of the cortical thickness; S4: primordium length exceeding 1/2 of the cortical thickness; S5: primordium length exceeding 4/5 of the cortical thickness; S6: primordium completely penetrating the cortex. Scale bar = 50 µm. (B) Frequency distribution (%) of LRP at each developmental stage (S1–S6) in B73 and *dlr9-1*. A total of 50 lateral root primordia were scored for each genotype. (C) Cross-sections (left) and longitudinal sections (right) of root tissues showing LRP morphology in B73 and *dlr9-1*. Scale bar = 50 µm. (D) Quantitative comparison of LRP area (× 10³ µm²) between B73 and *dlr9-1*. Data were obtained from 50 lateral root primordia per genotype. Values are presented as mean ± SD. ** indicates a statistically significant difference as determined by Student's *t*-test (*p* < 0.01).

**Figure S3.** Genetic segregation of root phenotypes in *dlr9-1*. (A) Representative seedling phenotypes of (from left to right): the wild-type inbred line B73, the *dlr9-1*, their F_1_ hybrid (B73 × *dlr9-1*), and the segregating F_2_ population displaying either a wild-type (B73-like) or mutant (*dlr9-1-*like) phenotype. Scale bar = 2 cm. (B-C) Statistical analysis of lateral root number (B) and primary root length (C) for the genotypes shown in (A). n = 5, Statistical significance was determined using Student's *t*‐test, ***p* < 0.01.

**Figure S4.** Whole-genome and chromosome 1 SNP index plots from MutMap analysis of *dlr9-1*. (A) Single nucleotide polymorphism (SNP) index plots of *dlr9-1* for whole genome generated by MutMap analysis. (B) SNP index plots of *dlr9-1* for chromosome 1. (A, B) Dots indicate SNP index values at a given SNP position. Black lines represent the sliding window average SNP index values of the 0.1Mb interval with 100 kb step size. Chromosomal regions shaded gray indicate the genomic regions presumably harboring the causal mutations.

**>chromosome_1:301299387:+:RefGen_v4.32**

TTCTAACATAGCAACATGACCACACATGATCTGATCTGTATATAAAGCTGTACGAAATGTACCGAGTTCTATAAGCTTCTTTACAAGAAAAACGGCATATTTCTTGCAAGAAAGGGTAAGCAAATGTGGCTGTAGAACATCAAAGATAGCATCCCTCTCTGACTCCGAGCACCACTTTACACATGTCTGCCAAACGACAGTGAGCAACTGATCATCAAAAGCCAGGACACAGGCAACATAACAAAAAACGCAGCAGATTGACAAATACTGTGTGTGCTAAGCTTACTTGAAGAACACGTGCAGTAACATGTGATGTGGCAATGTCCAAATATTTCCCATCCATTTTATGGATAGCCTGACTTACTGCCCTGGAATGGTGACAATTATGTCAATGTATGAATGCAAACATAAAGATACAAGACTGACCCTATTGCTTACTTGGACCTCTCCTCTTTGCTCACATCGTGACATCTCATCTTTTCCCACAATTTCGTTAGTTC**T**TGGCCAGTATTCAGGGTCAAACAGAGCAAACAAGCATTGAATTTAGCAACCTGACGAGTTTACTGTCACCACAAACATCAGTCAAGGTTGAAGAACTTGTTGGTGTTGATGATCTTAGTGGAGGCAACACCCAAATTCAAAATCGAAAAAGGCAAAAATCAGGCTTGGATAGTAGAGCACATAATAGCAATCATCAAAAGAACCGTTCAATAAGTAAGTAGTGCAAGCAAAACCTAGTTGCAGAAACTGGCTCTCTGGACATATGACTACATACAGGCTGATGTGTTTAACATAAACTCTGTCCACATCGGACCGATCCATCATAGTGCAAGTCAGGATGCAGCTTGTCAGCAAAAGTATTACAACAAGAATCTTAATTTATAATATGACCTGAACATAGGTGGTGCAAGCCCCAAACTGGAACATATGGTGCGGAAAAACAGCACTGAAGTACCTTCTCAAGGCTGTAATGCTGCCTCCTCTTCATCTTCCTGGCCTCC

**Figure S5.** The 500 bp upstream and downstream sequences flanking the mutation site of *Zm00001d034750* in *dlr9-2.* Red characters represent *dlr9-2* mutation sites (C-T). The green-filled and underlined regions represent exons 3/2 and intron 2, respectively.

**Figure S6.** Alternative splicing of *ZmDLR9* gene in *dlr9-2*. (A) Schematic representation of the *ZmDLR9* gene structure, showing exons (purple boxes) and introns (black lines). The location of the *dlr9-2* point mutation in intron 2 is indicated by a red arrow. Primer binding sites for the molecular assay are marked: forward primer (F) in exon 2 and reverse primer (R) spanning the junction of exons 3 and 4. Scale bar = 1 kb. (B) Sanger sequencing chromatograms of genomic DNA from B73 and *dlr9-2*. The black arrow highlights the G-to-A transversion at the mutation site in intron 2. (C) Agarose gel electrophoresis of RT-PCR products amplified from cDNA of B73 and *dlr9-2* homozygous using primers F and R. The wild-type product is approximately 120 bp, while the *dlr9-2* produces a larger product of approximately 570 bp (indicated by arrows). Lane M, DNA size marker. (D) Sequencing chromatograms of the gel-purified PCR products from (C), confirming the nucleotide sequences of the wild-type and mutant *ZmDLR9* transcripts. (E) Model illustrating the splicing pattern of *ZmDLR9* in B73 and *dlr9-2*. In B73, intron 2 (red dashed line) is correctly spliced out. The *dlr9-2* (underlined in the sequence) disrupts the splicing donor or acceptor site, leading to the retention of intron 2 in the mature mRNA, which accounts for the larger transcript size observed in (C).

**Figure S7.** Genetic allelism test and root phenotypes of *dlr9* alleles. (A) Schematic of the genetic cross to test allelism between the *dlr9-1* and *dlr9-2* alleles. A homozygous *dlr9-1* (*ZmDLR9^dlr9-1/dlr9-1^*) plant was crossed as a female parent with a heterozygous *dlr9-2*(*ZmDLR9^dlr9-2/+^*) plant as the male parent. The resulting F_1_ progeny segregated into two genotypic classes: 46 individuals were heterozygous for *dlr9-1*(*ZmDLR9^dlr9-1/+^*), and 56 individuals were compound heterozygotes carrying both mutant alleles (*ZmDLR9^dlr9-1/dlr9-2^*). The 1:1 segregation ratio confirms that *dlr9-1* and *dlr9-2* are allelic mutations. (B) Root phenotypes of the parental lines and the segregating F_1_ progeny: *ZmDLR9^dlr9-1/+^* and *ZmDLR9^dlr9-1/dlr9-2^*. Images correspond to magnified views of the seedlings presented in Figure 2, Scale bar = 5 cm. (C-D) Primary root length (C) and lateral root number (D) of F_1_ progeny and parental seedlings shown in (B). n = 3, statistical significance was determined using Student's *t*‐test, ***p* < 0.01. (E) Genotype identification of seedlings by allele test. Genotypes at mutation sites in the F_1_ progeny of the allelism test.

**Figure S8.** Phylogenetic analysis of APUM protein family in maize and *Arabidopsis*. The evolutionary tree was constructed using the neighbor-joining method, showing relationships among 44 APUM domain-containing proteins from maize and *Arabidopsis* (prefixed with APUM number). ZmDLR9 (Zm00001d034749_P003), is marked with red star. Bootstrap values (percentages from 1000 replicates) are indicated at nodes. The scale bar (0.20) represents number of amino acid substitutions per site. ZmDLR9 clusters with high bootstrap support (88%) in a clade containing At3g16810 (APUM24), suggesting that ZmDLR9 is a member of APUM24 subfamily. This analysis provides insight into evolutionary conservation and divergence of APUM protein family.

**Figure S9.** Sequence alignment and conservation domain analysis of ZmDLR9. (A) Multiple sequence alignment of the ZmDLR9_P003 protein (encoded by *Zm00001d034749_T003*) from maize with its orthologous proteins in other plant species, including the monocots sorghum (*Sorghum bicolor*; XP_002463569.1), foxtail millet (*Setaria italica*; XP_004981175.1), and rice (*Oryza sativa Japonica Group*; XP_015631977.1), and the dicots *Arabidopsis thaliana* (AT3G16810), tobacco (*Nicotiana tabacum*; XP_075085648.1), and *Populus euphratica* (XP_011011878.1). Identical residues are highlighted in pink, residues with ≥80% similarity in blue, and residues with ≥60% similarity in yellow. The red arrow labeled *dlr9-1* indicates the specific site of the *dlr9-1* mutation. The alignment reveals highly conserved regions across both monocot and dicot lineages, underscoring the functional importance of these domains. Variable regions correspond to species-specific sequence divergence. The number of aligned residues for each protein is indicated on the right. (B) Comparative domain architecture of ZmDLR9 across the plant species. Protein domain structures of homologous pumilio-containing proteins in seven plant species: *Zea mays*, *Sorghum bicolor*, *Setaria italica*, *Oryza sativa*, *Arabidopsis thaliana*, *Nicotiana tabacum*, *Populus euphratica*. All proteins share a conserved pumilio RNA-binding domain (highlighted in blue), indicating functional conservation across species. Protein lengths are drawn to scale (amino acid scale bar shown). Domain positions were predicted using SMART.

**Figure S10.** Analysis of *ZmDLR9* transcripts. Genome browser views showing the abundances of *ZmDLR9* transcripts (T001 to T007) in RNA‑seq profiles of B73. The upper scale indicates the genomic position on chromosome 1. The y‑axis represents normalized read counts (RPKM/TPM) from the genomic region. The lower schematic diagram shows the exon–intron structures of the seven transcript isoforms (*ZmDLR9_T001* to *ZmDLR9_T007*). Red Arrows and red dashed lines indicate that *ZmDLR9_T007* is the dominant transcript in maize roots.

**Figure S11.** Heterologous expression and functional validation of ZmDLR9 in *Arabidopsis.* (A) Exon-intron structure of *APUM24* showing the T-DNA insertion sites. The primers used for genotyping are indicated as arrows (Table S8). (B) Schematic diagram of heterologous expression vector of *ZmDLR9* gene. (C) Genotyping using PCR amplification of genomic DNA in Col-0, *apum24-2* and *apum24-2* complemented with the UBQ:ZmDLR9 construct (Comp-1 and Comp-2). (D) Phenotype of roots of Col-0, *apum24-2*, Comp-1 and Comp-2. Scale bar = 1 cm. (E-G) Primary root length (E), lateral root number (F), and Morphology (G) in Col-0, *apum24*, Comp-1 and Comp-2. Scale bar = 2 cm. (H) Venation phenotypes in three-week-old *Arabidopsis* seedlings. Cotyledon venation (upper panel) and first true leaf venation (lower panel) are shown for Col-0, *apum24*, Comp-1 and Comp-2. Scale bar = 2 mm.

**Figure S12.** Population nucleotide diversity analysis of *ZmDLR9* in maize. (A) Genome‑wide nucleotide diversity (π) profiles of the *ZmDLR9* genomic region (including 5 kb upstream and 5 kb downstream) in three maize populations: improved varieties (yellow line), traditional landraces (green line), and the wild progenitor *Zea mays* ssp. *parviglumis* (blue line). Population genetic parameters were calculated using a 10‑SNP sliding window based on data retrieved from the MaizeSNPDB database (http://150.109.59.144:3838/MaizeSNPDB/). (B) Nucleotide diversity (π, top panel), Tajima’s D statistics (middle panel), and Weir & Cockerham’s *F_ST_* (bottom panel) across the *ZmDLR9* and its 5‑kb flanking regions on chromosome 1 chr1: 302747203–302763279, based on the B73_RefGen_V5 maize reference genome). The light‑green shaded region indicates the *ZmDLR9* gene locus (chromosome 1: 302,752,203–302,758,279). At the bottom is the gene model of *ZmDLR9*, where blue boxes denote exons, black horizontal lines represent introns, and the orange triangle indicates the transcription direction.
